# Important Developments in Romanian Propolis Research

**DOI:** 10.1155/2013/159392

**Published:** 2013-05-30

**Authors:** Liviu Al Mărghitaş, Daniel S. Dezmirean, Otilia Bobiş

**Affiliations:** Faculty of Animal Breeding and Biotechnology, University of Agricultural Sciences and Veterinary Medicine Cluj-Napoca, Mănăştur Street 3-5, 400372 Cluj-Napoca, Romania

## Abstract

The most important developments in propolis analysis and pharmacological properties are discussed. In order to help in the Romanian propolis standardization, different methodologies for chemical composition analysis (UV-VIS, HP-TLC, and HPLC-DAD) are reviewed using new approaches and software (fuzzy divisive hierarchical clustering approach and ChromQuest software) and compared with international studies made until now in propolis research. Practical applications of Romanian propolis in medicinal therapy and cosmetics are reviewed, and quality criteria for further standardization are proposed.

## 1. Introduction

Propolis is a natural material, sticky and resinous, collected by bees from different tree buds and exudates found on plant wounds, mixed with own substances like enzymes, and transformed in order to be used for sealing different holes in the hive, to clean the comb cells or to hygienize the entire hive [1–3]. Bees produce propolis as a means of defense against microbes, moulds, and for “embalming” the intruders that may enter into the hive, preventing their putrefaction [1, 4]. 

Beside its uses to seal and clean the hive, propolis may be called a “chemical weapon” of the bees against human pathogen microorganisms and viruses [5–13], as well as bee pathogens [14–17]. 

Due to the popularity of using propolis in medicine and other domains, thousands of studies were developed all over the world, studying the chemical composition, functions, or different properties of propolis extracts. More than 2700 articles and 250 books were published in Elsevier journals or publishing house (http://www.elsevier.com/), more than 2000 articles in Medline (http://www.ncbi.nih.gov/), and more than 1500 studies in Wiley-Blackwell database (http://www.onlinelibrary.wiley.com/).

Most of the current literature concerning propolis is focused on determining the chemical composition and biological activity of propolis, beside determining the botanical and geographical origins, by analyzing comparatively the resins from which propolis is derived [4, 6, 18–20], but less studies have examined the antimicrobial properties of propolis against bee pathogens or honey bee immune responses [14, 17, 21, 22]. 

The present study aimed to review the publications upon Romanian propolis with respect to composition, bioavailability, and biological properties. 

## 2. Propolis Origin 

Scientific studies demonstrate that propolis components came from three distinctive sources:

- vegetal: plant exudates collected by the bees resins secreted by the buds of poplar, pine, birch, chestnut, maple [3, 18, 23, 24], and lipophilic substances secreted by plant wounds, resins, or gums [25–29];
- animal: substances secreted by the bees (wax, saliva) [3, 29, 30];
- incidental materials introduced during propolis production (pollen, nectar or honey) [3, 30–33].

Depending on the plant source collected by the bees, propolis color may vary from golden yellow, to red, green, or dark brown.

The complex chemical composition of propolis is due to the plant material used as the raw material by the bees, reason also for the wide range of compounds (in number and quantity) that are found in propolis from different parts of the world. Scientific studies on the chemical composition of propolis and different tree or other plant species from the surroundings of the collection places of propolis samples indicate the relation between the plant material and propolis samples from the same region: *Populus nigra*, *Populus italic*, and  *Populus tremula* (Bulgaria and Mongolia) [34, 35], *Betula*, *Populus*, *Pinus*, *Acacia*, and *Aesculus* (Hungary) [36], *Betula*, *Pinus,* and *Salix* (Poland) [37], deciduous trees and meadows (Lithuania) [38], *Populus* and *Betula* (Russia) [39], *Populus trichocarpa* and *Populus tremuloides* (Canada) [40], *Clusia minor* and *Clusia major* (Venezuela) [41–43], and *Baccharis* spp. (Brazil) [25, 44, 45]. 

Studies on chemical composition of propolis from different geographical origins show that characteristic classes of compounds for different geographical origins also correlated with plant source [10, 18, 46]. Birtaş-Gagea [46] made a detailed study for footprinting different Romanian plant sources extracts (poplar, birch, willow, and pine buds) compared with propolis extract, revealing that secondary metabolites from the mentioned plant sources, especially poplar, are found also in Romanian propolis samples. 

## 3. Early Researches in Romanian Propolis

As most of the propolis samples from Central and Eastern Europe, Romanian propolis plant sources are resins secreted by buds of *Populus nigra, Quercus, Aesculus hippocastanum, Ulmus, Picea, *and* Fraxinus*. Romanian poplar buds have been analyzed [47] for their flavonoid content using thin layer chromatography, and comparative studies were made with propolis extracts [48]. This showed that both contained the same pattern of flavonoids: chrysin, tectochrysin, pinocembrin, galangin, kaempferol, apigenin, and quercetol. For most of the compounds, the levels found in propolis were slightly higher than in poplar buds. The total flavonoid content was slightly higher in the poplar buds extract, due to the presence of beeswax, pollen, and other impurities in propolis. Methanolic extracts of *Populus nigra*, *Betula pendula*, *Salix alba,* and *Pinus* were analyzed [49], showing that these plant sources were used by the bees for propolis production. 

Many research groups have found evidence of biological activities of Romanian propolis extract. Early studies were focused on the effects of certain blood constituents and lymphatic system [50, 51], hepatotoxic and hepatoprotector effects [52, 53], and antibacterial, antiviral, and anti-inflammatory effect [54–59]. Many studies were made on the action of a standardized propolis extract (SPE) on rat liver [60–62]. 

Propolis extracts were also investigated for their action in agriculture. Different studies were focused on the germinative effect in different plant seeds [63–65] and vine [66]. Different water extracts of propolis were used as germination substrates and the stimulatory effect upon germination was established [67]. Onion (*Allium cepa* L.), linseed (*Linum usitatissimum *L.), wheat (*Triticum* sp.), and oat (*Avena sativa*) were monitored during 24 and 112 hours in substrates containing different dilutions of propolis aqueous solution, establishing daily germination percentages. Seeds of elegant zinnia (*Zinnia elegance*), Mexican marigold (*Tagetes erecta*), pot marigold (*Calendula officinalis*), strawflower (*Helichrysum bracteatum*), and marvel of Peru (*Mirabilis jalapa*) were stimulated in their germination capacity by 0.5% aqueous solution of propolis, compared with control groups. Vine layers for shoot formation and root striking, into water solutions of propolis in different concentrations, were followed for their time bill and bud-breaking, moment of advent of shoots, speed of growth, and average number of roots/layers. Low concentrations of propolis solutions (0.1%; 0.05% and 0.01%) present a stimulation of shoot formation and rooting.

*In vitro* studies of propolis extracts on different plant tissue micropropagation (carnation, onion) were performed, and interesting results were obtained [68–72]. Beside the stimulation of growth for *in vitro* cultivation of the plants, using propolis in the cultivation media, no infection appears in the culture vessels, with propolis playing a double role of stimulant and antiseptic. 

## 4. Chemical Composition 

Propolis is one of the most studied Romanian bee products. For chemical composition analysis, best extraction procedures were developed and comparatively assessed  [49, 73–75]. Solid-liquid extraction with different ethanol concentrations was performed in reflux condenser for one hour at 65°C or at room temperature for different periods of time (4–6 days) [49, 76] and compared with water extracts of propolis with respect to biologically active compounds extracted [77]. Detailed reports and Ph.D. degree theses on chemical composition [46, 78–86] and plant origin from bud exudates of *Populus* spp. and other species [47–49, 84, 87] were made and the common conclusion was that Romanian propolis possessed high amounts of biologically active compounds from the classes of phenolic acids and different classes of flavonoids (flavones/flavonols, flavanones/dihydroflavonols, or other phenols) [16, 46, 49, 85] and can be subjected to other “validated” methods for European poplar type propolis [88]. 

A simple method for total flavonoid determination was developed by Tămaş [89]. Different complementary colorimetric methods are required for total flavonoid determination, as aluminium chloride reacts only with flavone/flavonols and 2,4-dinitriphenylhydrazine reacts with flavanones/flavanonols [90]. This simple method suggests zirconium reagent for total flavonoid determination. The method is simple, reliable, and fast requiring only one reagent which reacts with all specific flavonoid compounds, estimating their total content. The method was used and verified by different research groups in Romania [84, 85]. 

For the first time a study on Romanian propolis analysis was made using reflectance spectroscopy and chemometric treatment of digitized spectra of solid propolis samples [91] and demonstrated that the raw samples of propolis carry valuable information about their origin analyzing the color, physical, and other chemical constituents. Cluster analysis (CA), principal component analysis (PCA), and linear discriminant analysis (LDA) were successfully applied to spectroscopic and score matrices. Having different botanical origin, propolis exhibits different colors (light orange, dark with reddish tint, dark with green tint etc.) and different UV-spectra. Based on the difference in color. A methodology of sampling technique and data analysis using chemometric methods applied on reflectance UV-spectra analysis of solid samples, was developed for the first time, based only on the difference in color, without any sample extraction, solvent use or sophisticated equipment needed.

Another simple method in discrimination and authentication of propolis samples is based on fuzzy clustering of thin layer chromatographic data via image analysis [86]. Known concentrations of propolis ethanolic extracts were applied on HP-TLC silica gel 60 precoated plates and eluted with toluene-ethyl acetate-formic acid (30 : 12 : 5) and developed with 0.2% diphenylboryloxyethylamine and 4% polyethyleneglycol. The fluorescent images were processed using TLC Analyzer software [92]. The results are in agreement with botanical origin and vegetation zone and conduct to the identification of two types of propolis: meadow area and forest area, with three subgroups in the first type and two subgroups in the second type. 

As mentioned before, propolis has a very complex chemical composition, depending on the flora from areas where it is collected. Romanian propolis proved to belong to the temperate zone propolis regarding the chemical composition. 

The research group of Coneac et al. [76] analyzing propolis from west side of Romania by HPLC-DAD identified and quantified caffeic acid, rutin, quercetin, apigenin, and chrysin, both in “hot” (reflux extraction) and “cold” extracts (maceration in different concentrations of ethanol). Using 20%, 60%, and 96% ethanol, different results were obtained: higher concentration of ethanol extracting more hydrophobic bioactive compounds (apigenin, kaempferol, and chrysin), compared with hydrophilic ones (caffeic acid and quercetin). 

**Figure 1:**
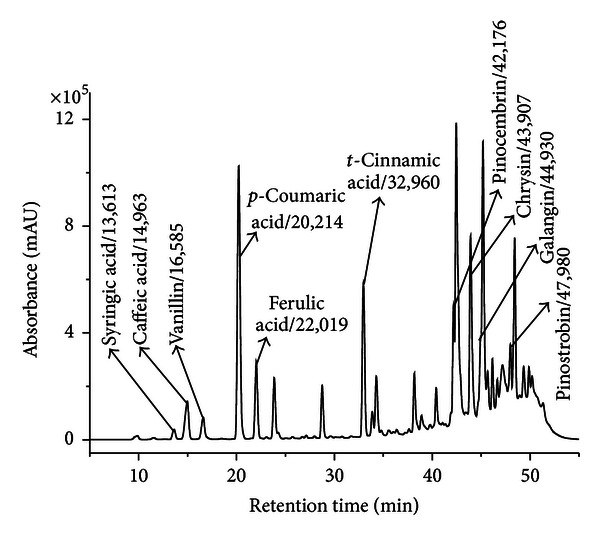
HPLC chromatogram of a Romanian propolis extract and the identified compounds/retention time [85].

The study of Mihai [85], investigating 53 propolis samples from Transylvania by means of HPLC-DAD, identified and quantified 10 compounds (phenolic acids and flavonoids) using external standard method. Calibration curves for each standard were made for exact quantification of compounds from propolis extracts. The identified compounds were syringic acid, caffeic acid, p-coumaric acid, ferulic acid, t-cinnamic acid, vanillin, pinocembrin, chrysin, galangin, and pinostrobin (Figure 1). All analyzed samples contain phenolic acids caffeic, p-coumaric, and ferulic and also chrysin. Most of the samples contain pinocembrin and galangin. Chrysin is the reference flavonoid in poplar propolis and was quantified in higher amounts in Transylvanian samples (1.6 mg/g propolis). The conclusion of the study was that also Transylvanian propolis belonged, to poplar type and using chrysin, galangin or pinocembrin in spectrophotometric determination of total flavonoids from propolis is correct, those compounds being present in almost all analyzed samples.

## 5. Antioxidant Activity

The antioxidant activity of Romanian propolis was studied and high biological activity was correlated with phenolic fraction of the extracts [76, 93–97]. 

Coneac et al. [76] suggest that using the DPPH method for radical scavenging activity measurement, relative absorbance *A%* must be calculated as the ratio between the absorbance at time *t* and initial absorbance, and the *A%* values used for comparis would be those from *t* time when *A%* became constant. Generally phenolic content is correlated with antioxidant activity, as the flavonoids are the major compounds responsible for this activity [95, 96]. 

An interesting work investigates the intimate molecular-level mechanisms of propolis extract, known for its antioxidant activity [93]. Electron paramagnetic resonance (EPR) detectable free radical signals are described for the first time in propolis extracts. The shape of these signals and the conditions in which they were obtained point to polyphenolic flavonoids as the sites of the radicals. An inverse correlation between antioxidant capacity and free radical signal intensity is shown. The free radical reactivity of propolis is also investigated by the effect that it exerts on the biologically relevant peroxide reactivity of hemoglobin. With this study, a new test of antioxidant activity from natural extracts is proposed, based on modulation of the ascorbate peroxidase activity of hemoglobin (HAPX). Obtained results correlate with those obtained by traditional methods such as 2,2-diphenyl-1-picrylhydrazyl (DPPH), or on 2,2′-azinobis(3-ethylbenzothiazoline-6-sulfonic acid (ABTS)). 

An effective measure of antioxidant capacity based on 2,2-diphenyl-1-picrylhydrazyl (DPPH) bleaching kinetic profiles has been developed using principal component analysis (PCA) [97]. The activity score as well as a related parameter, called *quercetin factor* (QF), was used to estimate antioxidant capacity of different propolis extracts based on the first principal component (which explains 98% of the total variance). Determination of the QF parameter requires less time and reagents than previous DPPH-based antioxidant capacity parameters but does require additional equipment. Additionally, UV-VIS and FT-IR spectroscopic analyses of propolis extracts have been performed and correlated to antioxidant capacity, to offer a spectroscopic and reagent-less rapid evaluation method of the antioxidant activity of biological samples. This test battery may be an interesting tool for antioxidant capacity, floral origin, and geographic location of propolis and other bee products.

## 6. Biological Properties

Antibacterial, antifungal, or antiviral activities were studied on propolis extracts of different geographical origin from Romania [16, 54–59, 98–103]. 

Three Gram-positive bacteria (*Staphylococcus aureus *ATCC 6538P, *Bacillus cereus *ATCC 14579, and *Listeria monocytogenes *ATCC 7644), two Gram-negative bacteria (*Escherichia coli *ATCC 10536 and *Pseudomonas aeruginosa *ATCC 27853), and one yeast strain (*Candida albicans *ATCC 90028) were used for testing the antibacterial activity of Romanian propolis [101]. Gram-positive bacteria were more sensitive to propolis extract, using inhibition zone method, while Gram-negative bacteria were more resistant. *Pseudomonas aeruginosa* was not inhibited by 5% ethanolic propolis solution. This bacterial strain is known for its resistance towards antibiotics also. Similar findings were obtained by Stan et al. [102] on *Pseudomonas aeruginosa* and *Staphylococcus intermedius*. 

Propolis samples from different geographical origin (different botanical origin) exhibit different antibacterial activity due to the interactions between the biologically active compounds present in the composition. The most recent study on Romanian propolis and its antibacterial activity on *Paenibacillus larvae*, the bacterial pathogen that causes American Foulbrood, a larval disease that can kill the honeybee colony, show very interesting results [16]. Different propolis extracts from Transilvania region (Romania) show significantly inhibition process of *P. larvae* tested *in vitro*. The extracts showed major differences in the content of total flavonoids (ranging from 2.4% to 16.4%) and the total polyphenols (ranging between 23.3% and 63.2%). The study revealed that it is not only the content of compounds in propolis which influences the strength of antimicrobial effects, but there is also a significant interaction effect among flavonoids of the propolis extracts. With this study, it was proposed that interaction effects among the various chemical compounds in propolis should be taken into account when considering the antibacterial effects against honeybee pathogens.

Romanian propolis was also used in different pharmaceutical and cosmetic formulations [77, 104–107]. A study made with propolis and lycopene extract nanoemulsion preparations confers better therapeutic effects than those of the conventional formulations, based on local control release of dozed form, for a longer period of time, which probably improves its efficiency and skin acceptance, meaning a better compliance [77]. This study is very interesting and useful for different cosmetic utilization of these nanoemulsions in skin's protective mechanisms against UVA radiation. The information obtained in the study of Butnariu and Giuchici [77] suggests that administration of propolis and lycopene aqueous extract nanoemulsion is safe and can be also useful for preclinical studies because of the high potential both regarding its efficiency (the analgesic effect) and therapeutic safety.

Propolis extracts previously characterized by HPLC were used to obtain micro/nanoparticles by encapsulation in β-cyclodextrin (solution method), and the complexes were analyzed again in order to evaluate the morphology and the dimensions of crystals and to evaluate the encapsulated biocompounds [104]. It was interesting to observe that the nanoparticles containing propolis extracts release the biologically active compounds more gradually, compared to propolis extract.

## 7. Quality Criteria for Propolis Standardization

In order to be accepted into the healthcare system, propolis needs chemical analysis performed by some standardized methods that may be applied all over the world. From this statement came the real problem for the researchers, because what can be observed from the existing studies is that propolis is very complex and different from one geographical region to another. This issue has an answer that propolis may be standardized, if different propolis types are formulated by their plant source and corresponding chemical profile. If regional standardization is taken into account, for poplar type propolis, which is the most studied and the best known propolis type, a battery of tests can be made to standardize the main compounds. 

**Figure 2:**
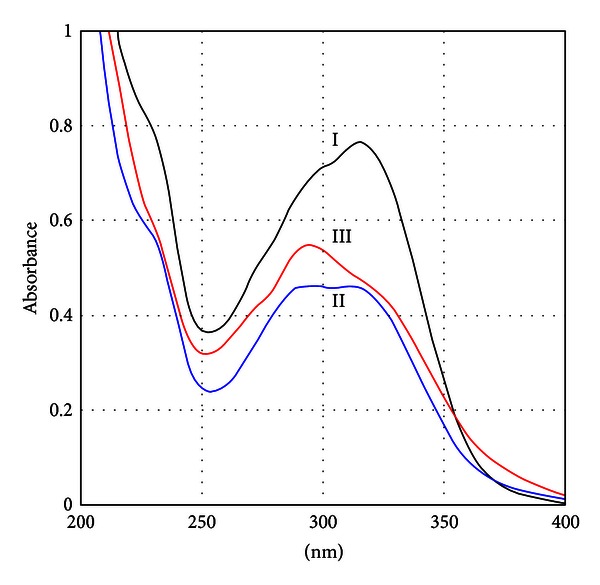
Overlapped UV-VIS spectra of different propolis extracts: I—propolis extract having *λ*_max⁡_ = 320 nm and total flavonoid content < 2%; II—propolis extract having a plateau as UV maxima between 320 and 395 nm and total flavonoid content approximately of 5%; III—propolis extract having *λ*_max⁡_ = 295 nm and total flavonoid content of 5%–8% [49].

Stan et al. [108] made a description of compositional characteristics for 56 Romanian propolis samples, proposing a battery of tests for future standardization. For covering all quality aspects, further work on more samples from all locations in Romania is needed in the future. Once a pattern of chemical constituents is well established (with high number of samples from all geographical regions in Romania and analyzed by laboratory validated methods), a standard for propolis analysis will be suitable for debating at national level. The most often techniques used generally for chemical analysis of propolis are spectrophotometric methods [23, 76, 80, 83, 84, 88, 90, 108], high performance liquid chromatography (HPLC) coupled with different detectors [10, 26, 27, 45, 49, 76, 78, 85, 109–112], and gas chromatography-mass spectrometry (GC-MS) [87, 112–115]. Interesting results were obtained [49] (Figure 2) in a simple spectrophotometric registration of UV-Visible spectra of different propolis extracts, correlated with total flavonoid content, where 3 types of propolis may be distinguished.

- Propolis spectra with *λ*_max⁡_ = 320 nm have total flavonoid content < 2%, and radical scavenging activity situated between 8% and 14%.
- Propolis spectra having a plateau as UV maxima between 320 and 395 nm exhibit a total flavonoid content approximately of 5% and radical scavenging activity of 14%–18%.
- Propolis spectra having *λ*_max⁡_ = 295 nm show total flavonoid content of 5%–8% and radical scavenging activity of more than 18%.

For quality purposes, propolis sampling procedures must include the type of collection method, because this may influence the quality of final product (tincture) [116]. Quality standards for bee products in general and propolis in particular must be for the benefit of producers, distributors, and consumers as well as. The present study shows that higher content of wax was determined in propolis samples obtained by scrapping the frames with hive tools than in samples obtained through propolis collector. Higher amounts of biologically active compounds were obtained from samples collected through propolis collector.

As a health promoting product, propolis quality must be always very high, even if there is still no certified system of quality control available worldwide [108]. Nevertheless, good apicultural practices should be applied by the beekeepers first and then the producers of different propolis-based products.

## 8. Conclusion

Propolis is a natural product with high potential for use in human consumption and medicinal uses. Quality control and chemical composition reveal that, generally, Romanian propolis is a high quality product, suitable for human consumption and uses in medicinal formulations. A high number of samples covering most of Romanian territory are analyzed with standardized methods available at this moment and a battery of tests for future standardization is proposed at this moment. The complex chemical composition of propolis requires further research in order to explain better its biological activities on different pathogens. Colony collapse disorder affects the bees all over the world, and self-medication of the bees is now of great interest. Initiated studies on using propolis for this purpose are in attention of different research groups from Romania, and original studies will be published in the near future.

## References

[1] Ghisalberti EL (1979). Propolis: a review. *Bee World*.

[2] Crane E (1988). *Beekeeping: Science, Practice and World Recourses*.

[3] Greenaway W, Scasbroock T, Whatley FR (1990). The composition and plant origins of propolis. A report of work at Oxford. *Bee World*.

[4] Bankova VS, De Castro SL, Marcucci MC (2000). Propolis: recent advances in chemistry and plant origin. *Apidologie*.

[5] Kujumgiev A, Tsvetkova I, Serkedjieva Y, Bankova V, Christov R, Popov S (1999). Antibacterial, antifungal and antiviral activity of propolis of different geographic origin. *Journal of Ethnopharmacology*.

[6] Banskota AH, Tezuka Y, Kadota S (2001). Recent progress in pharmacological research of propolis. *Phytotherapy Research*.

[7] Ito J, Chang FR, Wang HK (2001). Anti-AIDS agents. 48.^1^ Anti-HIV activity of moronic acid derivatives and the new melliferone-related triterpenoid isolated from Brazilian propolis. *Journal of Natural Products*.

[8] Chen C, Wu C, Shy H, Lin J (2003). Cytotoxic prenylflavanones from Taiwanese propolis. *Journal of Natural Products*.

[9] Bankova V (2005). Chemical diversity of propolis and the problem of standardization. *Journal of Ethnopharmacology*.

[10] Salomão K, Pereira PRS, Campos LC (2008). Brazilian propolis: correlation between chemical composition and antimicrobial activity. *Evidence-Based Complementary and Alternative Medicine*.

[11] Schnitzler P, Neuner A, Nolkemper S (2010). Antiviral activity and mode of action of propolis extracts and selected compounds. *Phytotherapy Research*.

[12] Watanabe MAE, Amarante MK, Conti BJ, Sforcin JM (2011). Cytotoxic constituents of propolis inducing anticancer effects: a review. *Journal of Pharmacy and Pharmacology*.

[13] Urushisaki T, Takemura T, Tazawa S (2011). Caffeoylquinic acids are major constituents with potent anti-influenza effects in brazilian green propolis water extract. *Evidence-Based Complementary and Alternative Medicine*.

[14] Antúnez K, Harriet J, Gende L, Maggi M, Eguaras M, Zunino P (2008). Efficacy of natural propolis extract in the control of American Foulbrood. *Veterinary Microbiology*.

[15] Bastos EMAF, Simone M, Jorge DM, Soares AEE, Spivak M (2008). In vitro study of the antimicrobial activity of Brazilian propolis against *Paenibacillus larvae*. *Journal of Invertebrate Pathology*.

[16] Mihai CM, Mârghitaş LA, Dezmirean DS, Chirilâ F, Moritz RFA, Schlüns H (2012). Interactions among flavonoids of propolis affect antibacterial activity against the honeybee pathogen *Paenibacillus larvae*. *Journal of Invertebrate Pathology*.

[17] Simone-Finstrom MD, Spivak M (2012). Increased resin collection after parasite challenge: a case of self-medication in honey bees?. *PLoS ONE*.

[18] Bankova V (2005). Recent trends and important developments in propolis research. *Evidence-Based Complementary and Alternative Medicine*.

[19] Bankova V (2009). Chemical diversity of propolis makes it a valuable source of new biologically active compounds. *Journal of ApiProduct and ApiMedical Science*.

[20] Miguel MG, Antunes MD (2011). Is propolis safe as an alternative medicine?. *Journal of Pharmacy & Bioallied Sciences*.

[21] Simone M, Evans JD, Spivak M (2009). Resin collection and social immunity in honey bees. *Evolution*.

[22] Moritz RFA, De Miranda J, Fries I, Le Conte Y, Neumann P, Paxton RJ (2010). Research strategies to improve honeybee health in Europe. *Apidologie*.

[23] Pietta PG, Gardana C, Pietta AM (2002). Analytical methods for quality control of propolis. *Fitoterapia*.

[24] Castaldo S, Capasso F (2002). Propolis, an old remedy used in modern medicine. *Fitoterapia*.

[25] Teixeira ÉW, Negri G, Meira RMSA, Message D, Salatino A (2005). Plant origin of green propolis: bee behavior, plant anatomy and chemistry. *Evidence-Based Complementary and Alternative Medicine*.

[26] Daugsch A, Moraes CS, Fort P, Park YK (2008). Brazilian red propolis—chemical composition and botanical origin. *Evidence-Based Complementary and Alternative Medicine*.

[27] Silva BB, Rosalen PL, Cury JA (2008). Chemical composition and botanical origin of red propolis, a new type of Brazilian propolis. *Evidence-Based Complementary and Alternative Medicine*.

[28] Bankova VS, Popova M, Trusheva B (2006). Plant solurces of propolis: an updatefrom a chemist’s point of view. *Natural Product Communications*.

[29] Gómez-Caravaca AM, Gómez-Romero M, Arráez-Román D, Segura-Carretero A, Fernández-Gutiérrez A (2006). Advances in the analysis of phenolic compounds in products derived from bees. *Journal of Pharmaceutical and Biomedical Analysis*.

[30] Burdock GA (1998). Review of the biological properties and toxicity of bee propolis (propolis). *Food and Chemical Toxicology*.

[31] Marcucci M (1995). Propolis: chemical composition, biological properties and therapeutic activity. *Apidologie*.

[32] Bankova VS, Christov RS, Tejera AD (1998). Lignans and other constituents of propolis from the canary islands. *Phytochemistry*.

[33] Valcic S, Montenegro G, Mujica A (1999). Phytochemical, morphological, and biological investigations of propolis from Central Chile. *Zeitschrift für Naturforschung*.

[34] Bankova V, Dyulgerov A, Popov S (1992). Propolis produced in Bulgaria and Mongolia: phenolic compounds and plant origin. *Apidologie*.

[35] Bankova V, Christov R, Popov S, Pureb O, Bocari G (1994). Volatile constituents of propolis. *Zeitschrift für Naturforschung C*.

[36] Nagy E, Papay V, Litkei G, Dinya Z, Farkas L, Gabor M, Kallay F (1985). Investigation of the chemical constituents, particularly the flavonoids components of propolis and *Populi gemma* by GC/MS method. *Flavonoids and Bioflavonoids*.

[37] Warakomska Z, Maciejewicz W (1992). Microscopic analysis of propolis from Polish regions. *Apidologie*.

[38] Ramanauskiene K, Savickas A, Inkeniene A (2009). Analysis of content of phenolic acids in Lithuanian propolis using high-performance liquid chromatography technique. *Medicina*.

[39] Bankova V, Popova M, Bogdanov S, Sabatini A (2002). Chemical composition of European propolis: expected and unexpected results. *Zeitschrift für Naturforschung C*.

[40] Christov R, Trusheva B, Popova M, Bankova V, Bertrand M (2006). Chemical composition of propolis from Canada, its antiradical activity and plant origin. *Natural Product Research*.

[41] Tomás-Barberán FA, García-Viguera C, Vit-Olivier P, Ferreres F, Tomás-Lorente F (1993). Phytochemical evidence for the botanical origin of tropical propolis from Venezuela. *Phytochemistry*.

[42] Cuesta-Rubio O, Frontana-Uribe BA, Ramírez-Apan T, Cárdenas J (2002). Polyisoprenylated benzophenones in Cuban propolis; biological activity of nemorosone. *Zeitschrift für Naturforschung*.

[43] Trusheva B, Popova M, Naydenski H, Tsvetkova I, Rodriguez JG, Bankova V (2004). New polyisoprenylated benzophenones from Venezuelan propolis. *Fitoterapia*.

[44] Marcucci MC, Bankova V (1999). Chemical composition, plant origin and biological activity of Brazilian propolis. *Current Topics in Phytochemistry*.

[45] Salatino A, Teixeira ÉW, Negri G, Message D (2005). Origin and chemical variation of Brazilian propolis. *Evidence-Based Complementary and Alternative Medicine*.

[46] Birtaş-Gagea GM (2011). *Footprint of bioactive compounds from poplar, birch, willow and pine buds, comparative with propolis [Ph.D. thesis]*.

[47] Tămaş M, Marinescu I, Ionescu F (1979). Flavonoids from poplar buds. *Studii Şi Cercetări Biochimice*.

[48] Marinescu I, Tămaş M (1980). Poplar buds—a source of propolis. *Apiacta*.

[49] Laslo L (2007). *Evaluation of propolis quality and authenticity markers [Ph.D. thesis]*.

[50] Giurgea R, Toma V, Popescu H, Polinicencu C (1981). Effects of standardized propolis extracts on certain blood constituents in chickens. *Clujul Medical*.

[51] Giurgea R, Popescu H, Polinicencu C (1982). Effects of standardized propolis extract on certain lymphatic organs and on immunologic reactions in chickens. *Clujul Medical*.

[52] Giurgea R, Rusu M, Polinicencu C (1984). Biochemical modifications in antihepatotoxic testing action of standardized propolis extract (EPS). *Flavonoids Symposium Bulletin, Cluj-Napoca*.

[53] Giurgea R, Rusu MA, Popescu H, Polinicencu C (1985). Biochemical modifications in carbone-tetrachloride intoxications and hepatoprotector effect of standardized propolis extract (SPE) in Wistar rats. *Clujul Medical*.

[54] Grecianu A, Enciu V (1976). Activity In vitro of propolis against bacterial strains of animal origin. *Institutul Ion Ionescu Dela Brad (Zootehnie, Medicina Veterinara)*.

[55] Eşanu V, Prahoveanu E, Crişan J, Ciocă A (1981). The effect of an aqueous propolis extract, of rutin and of a rutin-quercetin mixture on experimental influenza virus infection in mice. *Revue Roumaine de Medecine Serie de Virologie*.

[56] Mihail N, Giurgea R, Coprean D, Popescu H, Poliniceanu C (1984). Some data on anti-inflammatory action on a standardized propolis extract. *Revue Roumaine de Biologie*.

[57] Giurcăneanu F, Crişan I, Eşanu V, Cioaca V, Cajal N (1988). Traitement de l’herpes cutane et du zona zoster a l’aide de Nivcrisol-D. *Revue Roumaine de Medecine*.

[58] Olinescu R (1991). Antioxidant and anti-inflammatory action of propolis. *Studii Şi Cercetări Biochimice*.

[59] Dumitrescu M, Crişan I, Eşanu V (1993). Mechanism of the anti-herpetic activity of aqueous extract of propolis—II. Activity of lectins from the aqueous extract of propolis. *Romanian Journal of Virology*.

[60] Rusu M, Popescu H, Polinicencu C (1986). Histochemical aspects of standardized propolis extract (SPE) action upon intoxicated rat liver. *Flavonoids Symposium Bulletin, Cluj-Napoca*.

[61] Coprean D, Rusu M, Giurgea R (1986). The effect of the standardized propolis extract in experimentally intoxicated liver of rats. *Clujul Medical*.

[62] Giurgea R, Rusu MA, Coprean D, Popescu H, Polinicencu C (1987). Biochemical effects of standardized propolis extract (SPE) and of silymarin on the liver of ethyl alcohol intoxicated rats. *Agressologie*.

[63] Mărghitaş L, Mărghitaş M, Paşca I The effect of some aqueous propolis extract on the germination index at different seed types.

[64] Mărghitaş L, Mărghitaş M, Mudure E Researches regarding the germinativ effect of propolis.

[65] Dezmirean S, Mărghitaş LA, Dezmirean G, Szendrei I, Mudure E Researches regarding the effect of some bee products on plant germination.

[66] Mărghitaş L, Mărghitaş M, Pop N Researches regarding the influence of water extract of propolis on vegetative growth of Vitis vinifera vine.

[67] Mărghitaş L, Pamfil D, Bugnar L The hidrosoluble fraction from propolis influence upon the tissue culture “in vitro” in plants.

[68] Mărghitaş L, Pamfil D (1995). Water solution of propolis influence upon *Allium cepa L.* meristemes. *Bulletin of USAMV*.

[69] Mărghitaş L, Pamfil D The effect of aqueous propolis extract in carnation “in vitro” culture.

[70] Mărghitaş L, Pamfil D L’utilisation du propolis dans la culture *in vitro* chez les œillets.

[71] Mărghitaş L, Pamfil D Propolis utilization as carnation growth regulator.

[72] Dezmirean D, Mărghitaş LA, Pamfil DC (2003). Influence of honey and propolis on micropropagation of greenhouse carnation. *Bulletin of USAMV*.

[73] Mărghitaş L, Sabău A, Cornoiu I, Mudure E (1992). Researches on obtaining technology optimization in propolis. *Bulletin of USACN*.

[74] Dabija T (2006). *The improvement of extracting technology propolis and its usage in apiterapy [Ph.D. thesis]*.

[75] Laslo L, Mărghitaş L, Dezmirean D, Socaciu C (2005). Comparative analysis of propolis tincture. *Bulletin of USAMV*.

[76] Coneac G, Gafiţeanu E, Hădărugă DI (2008). Flavonoid contents of propolis from the West Side of Romania and correlation with the antioxidant activity. *Chemical Bulletin “Politehnica” University Timişoara*.

[77] Butnariu MV, Giuchici CV (2011). The use of some nanoemulsions based on aqueous propolis and lycopene extract in the skin’s protective mechanisms against UVA radiation. *Journal of Nanobiotechnology*.

[78] Croci AN, Cioroiu B, Lazar D, Corciova A, Ivanescu B, Lazar MI (2009). HPLC evaluation of phenolic and polyphenolic acids from propolis. *Farmacia*.

[79] Pop L, Mărghitaş L, Corradini D, Grego S, Laslo L (2005). Quantitative analysis of phenolic acids from Romanian propolis. *Bulletin of USAMV*.

[80] Mărghitaş AL, Dezmirean D, Laslo L, Moise A, Popescu O, Maghear O (2007). Validated method for estimation of total flavonoids in Romanian propolis. *Bulletin of USAMV*.

[81] Mărghitaş LA, Laslo L, Dezmirean D (2007). Radical scavenging activity of propolis. *Apiculture—From Science to Agribusiness and Apitherapy*.

[82] Mihai CM, Mărghitaş LA, Dezmirean D, Maghear O, Mărgăoan R, Stan Laslo L (2009). Transylvanian propolis from 7 counties—qualitative and quantitative analysis of phenolics. *Bulletin of USAMV*.

[83] Mihai CM, Mărghitaş LA, Bobiş O, Dezmirean D, Tămaş M (2010). Estimation of flavonoid content in propolis by two different colorimetric methods. *Scientific Papers: Animal Science and Biotechnologies, Timisoara*.

[84] Birtaş G, Mărghitaş LA, Dezmirean D, Stanciu OG, Tămaş M (2010). Spectrophotometric evaluation of flavonoid content of propolis and poplar buds by ZrOCl_2_ reagent. *Bulletin UASVM Animal Science and Biotechnologies*.

[85] Mihai CM (2011). *Evaluation of propolis quality from Transilvania with regards to standardization [Ph.D. thesis]*.

[86] Sârbu C, Moţ AC (2011). Ecosystem discrimination and fingerprinting of Romanian propolis by hierarchical fuzzy clustering and image analysis of TLC patterns. *Talanta*.

[87] Dobrinas S, Birghila S, Coatu V (2008). Assessment of polycyclic aromatic hydrocarbons in honey and propolis produced from various flowering trees and plants in Romania. *Journal of Food Composition and Analysis*.

[88] Popova M, Bankova V, Butovska D (2004). Validated methods for the quantification of biologically active constituents of poplar-type propolis. *Phytochemical Analysis*.

[89] Tămaş M (1979). Procedeu pentru determinarea cantitativă a flavonoidelor din propolis şi din preparate de propolis. *Brevet de Invenţie RSR*.

[90] Chang C, Yang M, Wen H, Chern J (2002). Estimation of total flavonoid content in propolis by two complementary colometric methods. *Journal of Food and Drug Analysis*.

[91] Moţ AC, Soponar F, Sârbu C (2010). Multivariate analysis of reflectance spectra from propolis: geographical variation in Romanian samples. *Talanta*.

[92] Soponar F, Moţ AC, Sârbu C (2008). Quantitative determination of some food dyes using digital processing of images obtained by thin-layer chromatography. *Journal of Chromatography A*.

[93] Moţ A, Damian G, Sarbu C, Silaghi-Dumitrescu R (2009). Redox reactivity in propolis: direct detection of free radicals in basic medium and interaction with hemoglobin. *Redox Report*.

[94] Mărghitaş LA, Dezmirean D, Moise A, Mihai CM, Stan Laslo L (2009). DPPH Method for evaluation of propolis antioxidant activity. *Bulletin of USAMV*.

[95] Mărghitaş LA, Dezmirean D, Mărgăoan R, Mihai CM, Ridley H, Farber M, Hull S Physico-chemical characterization and antioxidant activity of Transilvanian propolis.

[96] Mihai C, Mărghitaş LA, Dezmirean D, Bărnuţiu L (2011). Correlation between polyphenolic profile and antioxidant activity of propolis from Transilvania. *Scientific Papers Animal Science and Biotechnologies*.

[97] Moţ AC, Silaghi-Dumitrescu R, Sârbu C (2011). Rapid and effective evaluation of the antioxidant capacity of propolis extracts using DPPH bleaching kinetic profiles, FT-IR and UV-vis spectroscopic data. *Journal of Food Composition and Analysis*.

[98] Niculae M, Bobis O, Sandru C, Mărghitas L, Spinu M (2008). Antibacterial potential of romanian bee products and vegetal extractions against multiresistant *Staphylococcus aureus*. *Special Issue Apimondia Italia*.

[99] Mateescu C (1999). Propolis as therapeutic agents. *Honeybee Science*.

[100] Rindt IK, Spânu M, Niculae M, Szakacs BS, Bianu G, Laslo L (2009). The immunostimulatory activity of propolis from different origin. *Veterinary Medicine Scientific Papers*.

[101] Mărghitas LA, Mihai CM, Chirilă F, Dezmirean DS, Fiţ N (2010). The study of the antimicrobial activity of Transylvanian (Romanian) propolis. *Notulae Botanicae Horti Agrobotanici Cluj-Napoca*.

[102] Stan L, Niculae M, Mărghitaş LA, Spînu D Dezmirean M (2012). Antibacterial effect of Romanian propolis on *Pseudomonas aeruginosa* and *Staphylococcus intermidius*. *Planta Medica*.

[103] Salavastru CM, Nedelcu LE, Tiplica SG (2012). Management of leg ulcers in patients with chronic venous insufficiency: the experience of a Dermatology Clinic in Bucharest, Romania. *Dermatologic Therapy*.

[104] Coneac G, Gafiţanu E, Hădărugă NG (2008). Propolis extract/β-cyclodextrin nanoparticles: synthesis, physico-chemical, and multivariate analyses. *Journal of Agroalimentary Processes and Technologies*.

[105] Crişan I, Zaharia CN, Popovici F (1995). Natural propolis extract NIVCRISOL in the treatment of acute and chronic rhinopharyngitis in children. *Romanian Journal of Virology*.

[106] Ciulan V, Petruse C, Gârna AM (2008). Hemoleucogram changes on calves with neonatal diarrhea treated with propolis tincture, mint and bilberry extracts. *Bulletin of USAMV*.

[107] Siceanu A, Sapcaliu A, Rădoi I, Condor D, Căuia E, Pavel C (2008). The apiphytotherapy with Proactivator in the veterinary dermatology and surgery. *Lucrări Ştiinţifice-Zootehnie Şi Biotehnologii, USAMV Banatului Timişoara*.

[108] Stan L, Mărghitaş LA, Dezmirean D (2011). Quality criteria for propolis standardization. *Scientific Papers Animal Science and Biotechnologies*.

[109] Trusheva B, Popova M, Bankova V (2006). Bioactive constituents of Brazilian red propolis. *Evidence-Based Complementary and Alternative Medicine*.

[110] de Sousa JPB, Bueno PCP, Gregório LE (2007). A reliable quantitative method for the analysis of phenolic compounds in Brazilian propolis by reverse phase high performance liquid chromatography. *Journal of Separation Science*.

[111] Sha N, Huang H, Zhang J (2009). Simultaneous quantification of eight major bioactive phenolic compounds in Chinese propolis by high-performance liquid chromatography. *Natural Product Communications*.

[112] Chang R, Piló-Veloso D, Morais SAL, Nascimento EA (2008). Analysis of a Brazilian green propolis from *Baccharis dracunculifolia* by HPLC-APCI-MS and GC-MS. *Brazilian Journal of Pharmacognosy*.

[113] Pino JA, Marbot R, Delgado A, Zumárraga C, Sauri E (2006). Volatile constituents of propolis from honey bees and stingless bees from yucatan. *Journal of Essential Oil Research*.

[114] Márquez Hernández I, Cuesta-Rubio O, Campo Fernández M (2010). Studies on the constituents of yellow cuban propolis: GC-MS determination of triterpenoids and flavonoids. *Journal of Agricultural and Food Chemistry*.

[115] Popova MP, Graikou K, Chinou I, Bankova VS (2010). GC-MS profiling of diterpene compounds in mediterranean propolis from Greece. *Journal of Agricultural and Food Chemistry*.

[116] Stan L, Mărghitaş LA, Dezmirean D (2011). Influence of collection methods on propolis quality. *Bulletin of USAMV*.

